# Evaluating different adoption scenarios for TIL-therapy and the influence on its (early) cost-effectiveness

**DOI:** 10.1186/s12885-020-07166-9

**Published:** 2020-07-31

**Authors:** Melanie Lindenberg, Valesca Retèl, Maartje Rohaan, Joost van den Berg, John Haanen, Wim van Harten

**Affiliations:** 1 Division of Psychosocial Research and Epidemiology, the Netherlands Cancer Institute - Antoni van Leeuwenhoek hospital, Amsterdam, The Netherlands; 2 Department of Health Technology and Services Research, University of Twente, MB-HTSR, PO Box 217, 7500AE Enschede, The Netherlands; 3 Biotherapeutics Unit (BTU), The Netherlands Cancer Institute - Antoni van Leeuwenhoek hospital, Amsterdam, The Netherlands; 4 Department of Medical Oncology, The Netherlands Cancer Institute - Antoni van Leeuwenhoek hospital, Amsterdam, The Netherlands

**Keywords:** Tumor-infiltrating lymphocytes, Advanced melanoma, Implementation, Expert views, Health technology assessment

## Abstract

**Background:**

Treatment with tumor-Infiltrating Lymphocytes (TIL) is an innovative therapy for advanced melanoma with promising clinical phase I/II study results and likely beneficial cost-effectiveness. As a randomized controlled trial on the effectiveness of TIL therapy in advanced melanoma compared to ipilimumab is still ongoing, adoption of TIL therapy by the field is confronted with uncertainty. To deal with this, scenario drafting can be used to identify potential barriers and enables the subsequent anticipation on these barriers. This study aims to inform adoption decisions of TIL by evaluating various scenarios and evaluate their effect on the cost-effectiveness.

**Methods:**

First, 14 adoption scenarios for TIL-therapy were drafted using a Delphi approach with a group of involved experts. Second, the likelihood of the scenarios taking place within 5 years was surveyed among international experts using a web-based questionnaire. Third, based on the questionnaire results and recent literature, scenarios were labeled as being either “likely” or “-unlikely”. Finally, the cost-effectiveness of TIL treatment involving the “likely” scored scenarios was calculated.

**Results:**

Twenty-nine experts from 12 countries completed the questionnaire. The scenarios showed an average likelihood ranging from 29 to 58%, indicating that future developments of TIL-therapy were surrounded with quite some uncertainty. Eight of the 14 scenarios were labeled as “likely”. The net monetary benefit per patient is presented as a measure of cost-effectiveness, where a positive value means that a scenario is cost-effective. For six of these scenarios the cost-effectiveness was calculated: “Commercialization of TIL production” (the price was assumed to be 3 times the manufacturing costs in the academic setting) (−€51,550), “Pharmaceutical companies lowering the prices of ipilimumab” (€11,420), “Using TIL-therapy combined with ipilimumab” (−€10,840), “Automatic TIL production” (€22,670), “TIL more effective” (€23,270), “Less Interleukin-2” (€20,370).

**Conclusions:**

Incorporating possible future developments, TIL-therapy was calculated to be cost-effective compared to ipilimumab in the majority of “likely” scenarios. These scenarios could function as facilitators for adoption. Contrary, TIL therapy was expected to not be cost-effective when sold at commercial prices, or when combined with ipilimumab. These scenarios should be considered in the adoption decision as these may act as crucial barriers.

## Background

Over the past decade, the treatment landscape for advanced melanoma has greatly developed due to the introduction of checkpoint inhibitors and targeted therapies. This resulted in a rise of the 5-year survival rate from 10% [1] up to 52% [2] when using the most recent and promising treatment combination of nivolumab with ipilimumab.

Despite the improved clinical outcomes, a large group of patients still fail to respond or progress after initial response upon the available treatments. Therefore, the identification of additional treatment options for second-line treatment is of interest. Adoptive cell therapy with tumor-infiltrating lymphocytes (TIL) could be one of these additional treatment options. In TIL therapy, T cells residing in patient-specific tumor material are isolated and expanded ex vivo in a dedicated production facility and given back to the patient as a single intravenous infusion after a lymphodepleting non-myeloablative preparative regimen and subsequent treatment with interleukin-2 (IL-2). TIL treatment was introduced in small clinical trials in the ‘80s [3] and several research groups independently showed consistent objective response rates of 40–70% [4–6] and complete response rates of 10–25% [7], in subsequent small clinical phase I/II trials. However, this therapy has not yet been widely adopted. This can mainly be explained by the lack of phase III evidence of the clinical effectiveness of TIL therapy and the complex nature of this innovative cellular product (Advanced Therapy Medicinal Product (ATMP) of which clinical implementation is known to be challenging [8, 9].

Since October 2014, the Netherlands Cancer Institute (NKI) and the Herlev hospital in Denmark have been conducting the first randomized controlled trial (RCT) comparing TIL therapy to ipilimumab as a second-line treatment for advanced melanoma to evaluate its clinical and cost-effectiveness (NCT02278887). For the Netherlands, this trial is included in a Coverage with Evidence Development (CED) program for highly promising treatments [10]. This RCT aims to provide the evidence needed to widely adopt TIL therapy as a standard second-line treatment modality in advanced melanoma. As this trial is still ongoing, the decision for other centers and/or countries to adopt TIL therapy is surrounded by great uncertainty or is delayed. Especially delay could affect timely patient access when TIL therapy is proven to be effective, as the clinical implementation of TIL therapy is challenging and time-consuming [11].

In the framework of the CED program, a broad Technology Assessment (TA) is conducted to facilitate this clinical adoption of TIL therapy. Within this TA, an early cost-effectiveness analysis was conducted, showing that TIL therapy is cost-effective over ipilimumab as second-line treatment of advanced melanoma based on the currently available evidence [12]. Furthermore, a qualitative study was conducted evaluating barriers and facilitators in the clinical implementation of TIL therapy in light of an ATMP [11]. This study showed that its adoption can be influenced by many factors, such as the attitude of clinicians and patients due to the expected therapeutic risks and the rapidly evolving treatment field for advanced melanoma.

The current RCT conducted at the NKI and the final project in this TA aim to reduce the existing uncertainty surrounding the decision to clinically adopt TIL therapy as a second-line treatment for advanced melanoma. The objective of this paper is threefold. First, to evaluate various adoption scenarios related to TIL therapy and the treatment landscape of advanced melanoma (section 2.1). Second, evaluating the likelihood of these scenarios to occur within 5 years to identify potential barriers and facilitators for the adoption of TIL therapy (section 2.2), and third, to evaluate the cost-effectiveness of the likely adoption scenarios (section 2.3).

## Methods

In this study, we will often refer to “adoption scenarios”, which are one-sentence descriptions of potential developments that may affect the adoption of TIL therapy.

### Drafting adoption scenarios (Delphi methodology)

**Fig. 1:**
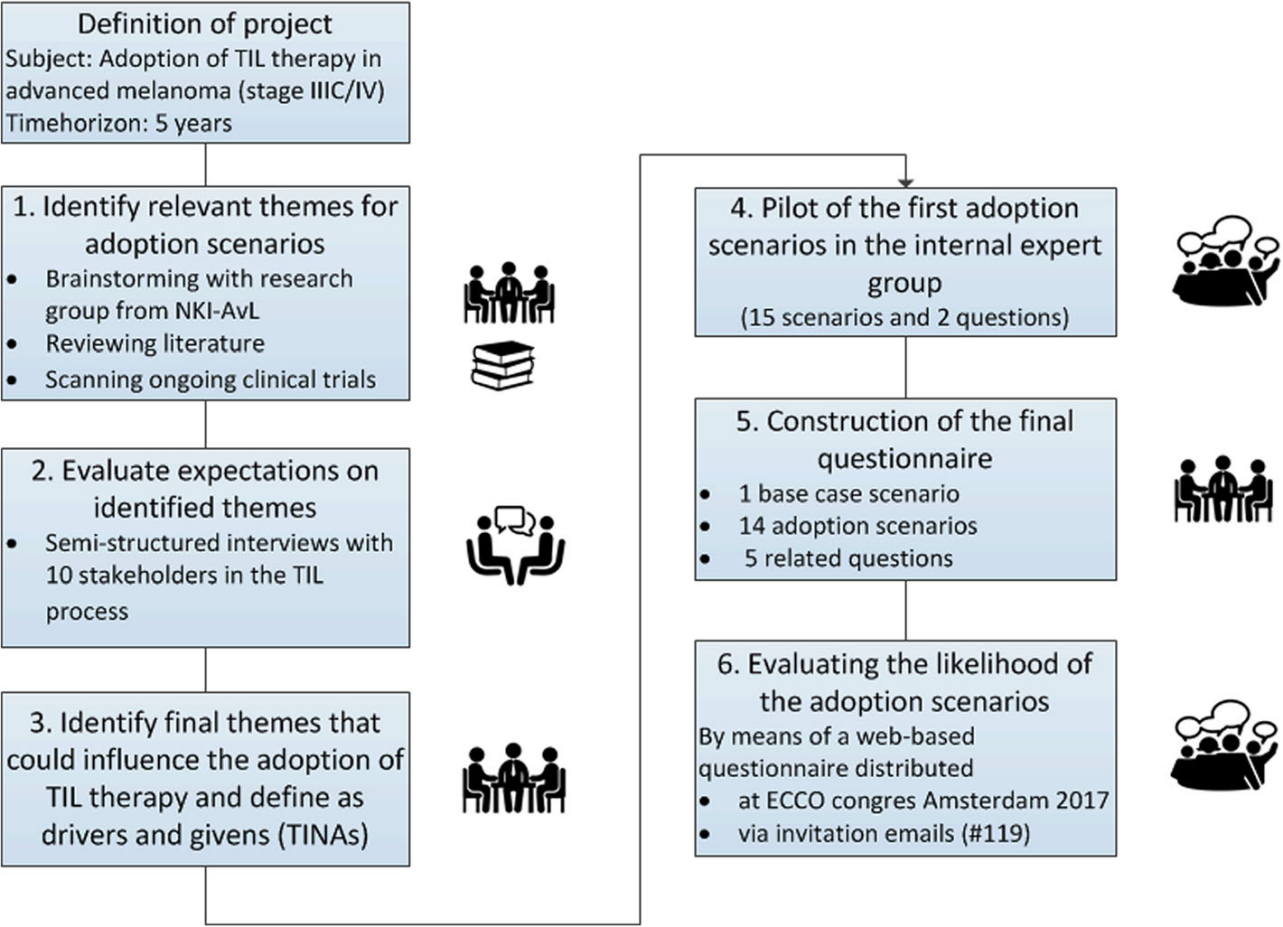
Schematic visualization of method and steps in drafting scenarios. Caption: This approach was based on the methods described by Shell international BV (2008) and Enserink and Hermans (2010) [13, 14]

A Delphi method was used to systematically generate consensus on themes related to the adoption of TIL therapy to subsequently incorporate these themes in the adoption scenarios. Figure 1 shows the six steps used to draft the scenarios [13, 14].

First, relevant themes that could influence the adoption of TIL therapy were identified through: brainstorming with internal experts, reviewing the literature on TIL therapy and research developments in treating advanced melanoma, and scanning ongoing clinical trials investigating TIL therapy. Second, the identified *themes* were discussed during semi-structured interviews with stakeholders in the TIL study process at the NKI to identify their expectations on these *themes* for the coming years [11]. They were allowed to add new *themes* and were specifically asked to describe likely “what if” scenarios for the coming five and 10 years [13]. The details on these semi-structured interviews are described in a previous publication [11]. In the third step, the results of the interviews were discussed with the direct research group (ML, VR, WvH), where the final *themes* were chosen to incorporate in the first (pilot) set of adoption scenarios. In step four, this first set of adoption *scenarios* (15 scenarios and two questions) was piloted in an expert group consisting of lab members, health insurers, clinicians, researchers, a representative of a patient association, a board member of the Dutch Immunotherapy Working Group for Oncology (WIN-O), and policy advisers. In the fifth step, the set was adapted according to their given feedback which resulted in the final set of scenarios. This set consisted of 15 adoption *scenarios* and 5 questions on, for example, minimal effectiveness, patients’ and clinicians’ attitudes towards TIL therapy (Table 1).

**Table 1:** Themes identified to draft scenarios and full description of scenarios

**Identified themes (result of step 2–4)**	
Less or even no interleukin-2, More automatic process, Attitude of clinicians, Costs of TIL, Take–over by a commercial party, Effectiveness TIL and others, Target population, Long term effectiveness, Attitude of patients, Unexpected clinical risks, Influence of pharmacy, Placement of TIL in treatment strategy	
**Name of scenario**	**Full description of scenarios**
Base case	If TIL shows better survival rates (at least 10% improvement) compared to ipilimumab, TIL will be implemented in specialized melanoma centers.
Competition	Competing (immuno)therapies are equal in costs but 10% more effective compared to TIL.
TIL more effective	The effectiveness of TIL has increased with 10% (clinically relevant) due to research developments.
Biomarker	A biomarker, being able to select patients for TIL, is available.
TCR therapy	TCR therapy dominates TIL treatment in advanced melanoma, regardless other treatment modalities.
Patients unconvinced	Patients prefer the competing therapies over TIL based on complete information on toxicities and effectiveness.
2nd line treatment	TIL is implemented as a second line treatment after anti PD1 inhibitors in metastatic melanoma.
3rd line treatment	TIL is implemented as a third line (last resort) treatment in metastatic melanoma.
Combination therapy	TIL is used in combination with other immune or personalized therapies (i.e. nivolumab or vemurafenib).
Clinicians unconvinced	Clinicians are not willing to implement TIL because of one of the previous stated reasons.
Low cost competition	If TIL turns out to be cost-effective, pharmaceutical companies will lower the prices of competing immunotherapies.
Influence by companies	Arrangements between pharmaceutical companies and hospitals and/or doctors, negatively affect patient selection for TIL therapy.
Less IL2 treatment	Additional interleukin-2 treatment after infusion of TIL is not be necessary anymore.
TIL production outsourced	Production of TIL is of interest for the pharmaceutical market and is outsourced by a commercial company.
Automatic TIL production	Production of TIL is less expensive (30% reduction) due to more automatic process steps.
**Questions**	
What would be the minimal effectiveness of TIL leading to accept TIL as a standard therapy for you? Expressed in one-year survival rate (%)?	
What would be the risk of developing other types of cancer such as lymphomas by activating the immune system by injecting TILs (%)?	
In which level do you agree with the following statement: TIL treatment provides significantly better quality of life compared to ipilimumab.	
Could you estimate the percentage of the eligible patients (metastatic melanoma patients) you think is aware of TIL therapy as a potential treatment (in %)	
What would be the main reason for clinicians to be unconvinced of introducing TIL therapy?	

### Estimating the likelihood of scenarios

The adoption scenarios and questions were included in a web-based questionnaire (Supplement 1) and were shared among a larger group of experts to evaluate the likelihood of the scenarios happening in the coming 5 years. To reach international clinical experts, flyers regarding the questionnaire were distributed at the congress of the European Cancer Congress Organization (ECCO) in Amsterdam (January 2017) after melanoma-related sessions. Additionally, the questionnaire was emailed to the scientific and clinical network of our internal experts, by which we invited 119 international experts; all were reminded after 1 month.

The questionnaire consisted of three parts. Part one introduced the TIL therapy and the RCT that is currently ongoing. Part two evaluated the characteristics of the respondent (years of experience with TIL therapy and years of experience with melanoma care, their position, and their self-reported level of expertise with TIL therapy) [15]. The third part contained the 15 adoption scenarios and the five questions, as listed in Table 1. In this part, the respondents indicated the likelihood of the scenarios occurring in the coming 5 years from 0 to 100%. 0% indicates that the scenario will not occur within 5 years, and 100% indicates that the scenario will occur within 5 years. This method is similar to the method used in a publication focusing on the adoption of Next Generation Sequencing [16]. Table 1 lists the names of the scenarios which are used in the following sections to refer to the specific scenarios.

### Calculating the cost-effectiveness

#### Selection of scenarios

As the likelihood of the 15 adoption scenarios (Results section 3.1) showed a lot of uncertainty, we followed several steps to label the scenarios as “likely” or “unlikely”. The process to select the “likely” scenarios to incorporate in the cost-effectiveness analysis is visualized in Supplement 2 and described in the section below.

To start, the mean likelihood of each scenario was evaluated. A scenario with a mean likelihood of ≥55% was labeled as “likely”. The scenarios that scored a likelihood < 55% were stratified in two ways; first, on the answers given to the level of expert by evaluating the results of the respondents that described themselves as “familiar” and “expert” (*n* = 23), and second for the level of experience evaluating the results from the respondents with ≥1 year experience with TIL therapy (*n* = 10). For the scenarios that still showed a score < 55%, a recent literature review was used [17]. When a topic related to the scenario was described in the review, the scenario was labeled as “likely”. Finally, if literature was also indecisive, the unlabeled scenarios were discussed and judged among experts (two clinicians, one technician, and a policy adviser) involved in the TIL study at the NKI, in which also the results on the five questions were discussed. Besides, the expert panel was asked to verify the likelihood of the scenarios labeled “likely” based on the cutoff value.

As it is plausible that several scenarios will take place at the same time, the same group of experts defined possible combinations of the “likely” scenarios. These were additionally incorporated in the cost-effectiveness model.

#### The base case model

A base case model is the original model used to evaluate the cost-effectiveness of an alternative treatment compared to the current standard of treatment using the best available evidence at that moment. In the current study, this is the cost-effectiveness model previously described by Retèl et al. (2018) [12]. This analysis was performed from a Dutch perspective evaluating TIL therapy in second-line treatment compared to its current standard of practice in second-line treatment, ipilimumab. A willingness to pay threshold of €80,000 per QALY gained was used. The model contained three health states: stable disease, progressive disease, and death (absorbing state). The time horizon was 10 years, reflecting an average lifetime time horizon of this patient group, with a cycle time of 1 year. Details on this model can be found in the original research paper [12] and Supplement 3. For clarity, we assumed that there would be no changes in costs and effects of TIL therapy and ipilimumab over the coming 5 years.

#### Incorporating the selected scenarios

The scenarios labeled as “likely” were incorporated in the cost-effectiveness model. With the experts (two clinicians, one technician, and a policy adviser) involved in the TIL study at the NKI, logical consequences were defined per scenario and were then translated to input parameters for the model. For some scenarios, an additional literature search was performed to feed the cost-effectiveness model. Although assumptions could be made for the efficacy of the scenario to use TIL therapy in the third line based on literature [5], no data or literature was found describing Progression-Free Survival and Overall Survival data of chemotherapy after progression on PD-1 inhibitors and CTL-4 antibodies, to serve as the comparator [18]. Therefore, this scenario wasn’t incorporated in the cost-effectiveness model. The scenario-specific input parameters, assumptions, and sources per scenario are listed in Table 2.

**Table 2:** Adapted input parameters for cost-effectiveness model per scenario

Adapted parameter	Initial deterministic value	Deterministic value	SE	Distribution	Source / Assumption
Scenario: “TIL more effective”					
PFS TIL	0.234	0.257	0.068	Beta	Assumption: 10% increase of survival rates as described in the scenario
OS TIL	0.412	0.453	0.046	Beta	
Scenario: “Combination therapy”					
PFS TIL	0.234	0.264	0.089	Beta	12mo PFS 4/13 patients [19] SE was kept the same as the initial model
OS TIL	0.412	0.499	0.098	Beta	12mo OS 9/13 patients [19] SE was kept the same as the initial model
Costs TIL	€ 62.000	€ 107.744	€13.743	Gamma	On average 2 times ipilimumab and administration costs and costs to anticipate on the side effects (€693.75 + €45,050) [19, 20].
Failure rate	0.10	0.10	0.015	Beta	1/13 received no TIL due to progression during TIL growth; 1 patient did not receive ipilimumab after TIL due to dose-limiting colitis [19]. Assumed to be similar as basecase model.
Scenario: “Low cost competition”					
Drug costs Ipilimumab	€ 90.100	€ 71.184	€9080	Gamma	Reduced price for ipilimumab in such a way that TIL is not cost-effective anymore with a willingness to pay threshold of 30.000. A reduction of 21%.
Scenario: “Less IL2 treatment”					
Total TIL costs	€ 62.000	€ 61.450	€ 7838	Gamma	Assuming the decrescendo regimen described by Andersen et al. 2016 6 vials of Aldesleukin (Novartis) [20] 550 euros reduced compared to the initial costs.
Utility decrements for side effects in providing TIL therapy due to toxicity	0.145	0.145	0.020	Beta	It was assumed to be the same as in the initial model because the availability of data on toxicity after a high or decrescendo dose scheme is limited.
PFS TIL	0.234	0.234	0.089	Beta	Assumed to be the same as no data shows that efficacy of TIL therapy decreased with a lowered dose IL2.
OS TIL	0.412	0.412	0.098	Beta	
Scenario: “TIL production outsourced”					
TIL production costs	€ 35.500	€ 106.500	€11.990	Gamma	Since no commercial price is available, we made an assumption based on expert opinion (WvH and JvB) that commercial costs of TIL are at least 3 times higher. Taking into account the necessary logistical arrangements and general costs when starting a biotech company
Scenario: “Automatic TIL production”					
TIL production costs	€ 35.500	€ 24.850	1268	Gamma	Assumption: 30% decrease of production costs as described in the scenario.

### Data analysis and visualization

The results of the scenarios incorporated in the cost-effectiveness model are expressed by the Incremental Cost-Effectiveness Ratio (ICER), Net Monetary Benefit (NMB), and the probability of TIL therapy being cost-effective. The ICER is a deterministic statistic calculated by dividing the difference in costs by the difference in Quality Adjusted Life Years (QALYs) for TIL therapy and Ipilimumab. An ICER, negative (less costly, more effective) and/or below a certain threshold (Willingness To Pay (WTP)), in this study €80,000, would mean that TIL therapy is favored over Ipilimumab. The WTP of €80,000 is the informal ceiling ratio in the Netherlands for diseases with the highest symptom burden [21]. As internationally different WTP thresholds are used, a second WTP threshold was used in evaluating the NMB: £30,000 (€34,821; April 2019), which is the WTP threshold used in the United Kingdom [22]. A two-way sensitivity analysis evaluates the effect of various levels of two parameters on the ICER. We varied the 1-year progression-free survival rate and the costs of TIL in a two-way sensitivity analysis.

Both NMB and probability of being cost-effective are probabilistic statistics in which uncertainty surrounding the input parameters is taken into account by randomly drawing parameter values from the parameter distributions, using Monte Carlo simulations with 1000 iterations. The NMB was calculated using the WTP ratios and the following formula per iteration: (incremental QALYs x WTP) - incremental costs. A mean NMB ≥ €0 indicates that TIL therapy is cost-effective compared to ipilimumab, given the chosen threshold.

To calculate the probability of TIL therapy being cost-effective, the NMB was calculated over different thresholds, ranging from €0 to €80,000 in steps of €1000. An NMB value of ≥€0 is cost-effective, which is indicated with 1, an NMB value <€0 is not cost-effective, which is indicated with 0. This was done for all the iterations in the Monte Carlo simulation per threshold. A mean of this binary value was calculated per threshold which shows the probability of TIL being cost-effective compared to ipilimumab at that threshold. Finally, the mean of these average probability scores gives the probability of TIL therapy being cost-effective in a WTP range of €0 - €80,000.

## Results

### Characteristics of the respondents

Twenty-nine respondents, mainly clinicians (76%; 24% other), completed the web-based questionnaire between January and October 2017. The majority of respondents originated from the Netherlands (*n* = 14), fifteen experts originated from other countries, namely Belgium, Denmark, Germany, Israel, Italy, Poland, Portugal, Spain, UK, the US, and Australia. Most respondents described themselves as familiar (52%), expert (28%), or a former expert (10%) with TIL therapy and had on average 2.7 years of experience with TIL treatment. Table 3 shows the characteristics of the respondents.

**Table 3:** Characteristics of the experts that participated in the scenario drafting questionnaire (*n* = 29)

Number of respondents	29 (100%)
Function	
Medical oncologist	22 (76%)
Director	3 (10%)
Head cell production	1 (3%)
Consultant	1 (3%)
Clinical and translational research	2 (7%)
Mean experience with melanoma, years *(range)*	16.38 *(1–35)*
Mean experience with TIL therapy, years *(range)*	2.72 *(0–20)*
Level of familiarity with TIL therapy	
Unfamiliar	0 (0%)
Accidentally familiar	3 (10%)
Familiar	15 (52%)
Former expert	3 (10%)
Expert	8 (28%)
Employed in:	
Australia	1 (3%)
Belgium	1 (3%)
Denmark	2 (7%)
Germany	3 (10%)
Israel	1 (3%)
Italy	1 (3%)
Netherlands	14 (48%)
Poland	1 (3%)
Portugal	1 (3%)
Spain	1 (3%)
UK	1 (3%)
US	1 (3%)
N/A	1 (3%)

### Likelihood of the scenarios

**Fig. 2:**
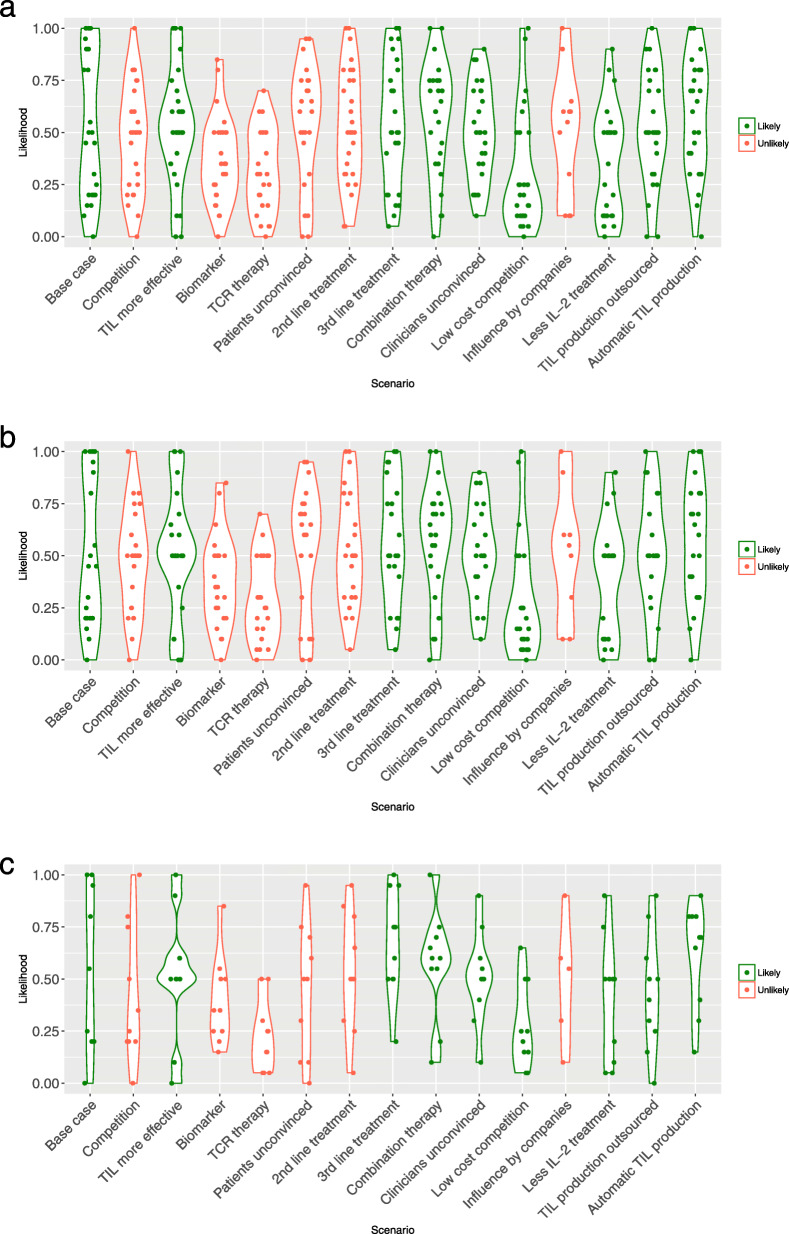
Likelihood of scenarios. Caption: This violin plot shows all observations from the survey in points. In addition, it shows the distribution of the likelihood per scenario by making the graph wider or smaller. When a number of observations are seen at the same likelihood percentage, the plot becomes wider. **a** shows the estimated likelihood of the future scenarios by all respondents (*n* = 29), **b** shows the estimated likelihood by only the respondents that evaluated themselves as an expert or familiar (*n* = 23), **c** shows the estimated likelihood by only the respondents with ≥1 year of experience with TIL therapy (*n* = 10). The colors green (“likely”) and red (“unlikely”) correspond to the final label of the scenarios that followed from the steps shown in Fig. 2 and according to the reasons stated in Supplement 5

The mean and median likelihood of each of the scenarios is presented in Table 4 and Fig. 2. Large variability was seen in the expected likelihood of the scenarios suggesting that respondents are uncertain about the future developments surrounding TIL therapy (Fig. 2). On average, most of the scenarios scored a likelihood of around 50% (46–54%). Two scenarios scored a likelihood of ≥55%: “Combination therapy” (57%) and “Automatic TIL production” (58%). Four scenarios were thought to be less likely: “Biomarker” (37%), “TCR therapy” (32%), “Low-cost competition” (30%), and “Less IL-2 treatment” (36%). Finally, the likelihood of the “Base case” in the coming 5 years was estimated at 54%. The results of the questions related to the adoption of TIL are listed in Supplement 6.

**Table 4:** The mean and median likelihood of each scenario

	*Mean likelihood (median)*		
	*All respondents (n = 29)*	*Only familiar and experts (n = 23)*	*≥ 1 year experience (n = 10)*
Base case scenario			
**“Base case”**	**54.3% (50%)**	**51.8 (45%)**	**55% (55%)**
“What if” scenarios			
“Competition”	46.4% (50%)	47.6% (50%)	42.5% (30%)
“TIL more effective”	51.9% (50%)	52.4% (50%)	52% (50%)
“Biomarker”	36.7% (35%)	38.3% (35%)	39.5% (35%)
“TCR therapy”	32.0% (30%)	29.3% (25%)	22.5% (20%)
“Patients unconvinced”	52.9% (60%)	53.6% (63%)	45% (50%)
“2nd line treatment”	52.8% (50%)	53.7% (50%)	53.5% (50%)
**“3rd line treatment”**	**54.5% (50%)**	**56.8% (50%)**	**67% (68%)**
**“Combination therapy”**	**57.3% (63%)**	**56.7% (60%)**	**57% (60%)**
“Clinicians unconvinced”	50.6% (50%)	51.6% (50%)	51% (50%)
“Low cost competition”	29.5% (20%)	28.7% (15%)	27.5% (23%)
“Influence by companies”	50.00% (58%)	51.7% (55%)	49% (55%)
“Less IL2 treatment”	35.9% (50.%)	39.1% (50%)	40.5% (50%)
“TIL production outsourced”	53.0% (50%)	51.5% (50%)	44% (45%)
**“Automatic TIL production”**	**58.4% (63%)**	**57.0% (60%)**	**62% (70%)**

The first column shows the likelihood by all respondents, the second column shows the likelihood judged by the respondents that judged themselves as expert and familiar and the third column shows the respondents having ≥1 year experience with TIL therapy. The scenarios displayed in bold were labelled as “likely” based on the evaluated likelihood (≥55% in one of these columns) (Fig. 2)

### Selected scenarios for incorporation in cost-effectiveness analysis

Using the cut-off value of ≥55%, “Combination therapy” and “Automatic TIL production” were labeled as “likely”. Using the stratified results based on the level of expertise, “Base case” and “3^rd^ line treatment” were also labeled as “likely” (Table 4). The expert panel verified the likelihood of those four scenarios. Based on the literature review (step four in Supplement 2), “TIL more effective” and “Less IL-2 treatment” were labeled as “likely” as several studies described potential opportunities to increase the effectiveness of TIL therapy and studies are investigating an IL-2 decreasing dose scheme to lower the intensity of the treatment [17]. The other scenarios or topics were not described in the recent literature review. The experts evaluated (step five) “Clinicians unconvinced”, “Low-cost competition”, “TIL production outsourced” as “likely” and the scenarios “Competition”, “Biomarker”, “TCR therapy”, “Patients unconvinced” and “Influence by companies” were labeled as “unlikely”. No scenario was solely labeled as “unlikely” based on the score from the survey. The arguments for labeling these scenarios as “likely” or “unlikely” are described in Supplement 5. As the base case scenario already evaluates the effect of using TIL therapy as a second-line therapy, the scenario: “2^nd^ line treatment” was not incorporated in the cost-effectiveness analysis because it would show the same results. Eventually, scenarios resulting in no implementation of TIL therapy e.g. “Patients unconvinced” and “Clinicians unconvinced”, regardless of their likelihood, were not incorporated in the cost-effectiveness model as this results in an analysis comparing ipilimumab to ipilimumab.

Additionally, the potential combinations of scenarios were drafted and incorporated in the cost-effectiveness model. Three combinations were made related to research developments including “TIL more effective”, “Automatic TIL production” and “TIL production outsourced”. Besides, three other combinations were defined incorporating the scenario “combination therapy”, and the scenarios: “Automatic TIL production”, “less IL-2” and “Low-priced competition”,

### Cost-effectiveness analysis

**Fig. 3:**
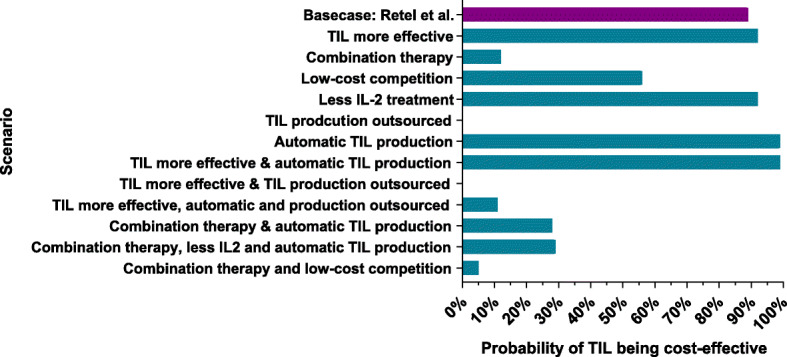
The probability of a scenario being cost-effective. Caption: Shows the probability of the different scenarios and the combinations of scenarios to become cost-effective when using a WTP threshold range of €0 to €80,000

**Fig. 4:**
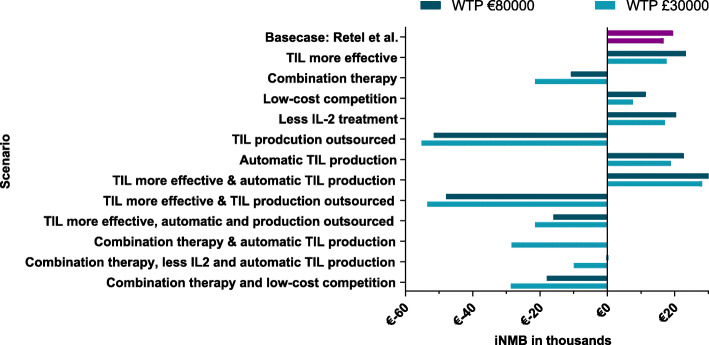
The incremental Net Monetary Benefit (iNMB). Caption: Shows the incremental Net Monetary Benefit (iNMB) for both the Dutch informal WTP threshold of €80,000 and for the WTP threshold that is mainly used in the United Kingdom of £30,000 (€34,821). A mean NMB ≥ €0 indicates that TIL therapy is cost-effective compared to ipilimumab given the chosen threshold

**Fig. 5:**
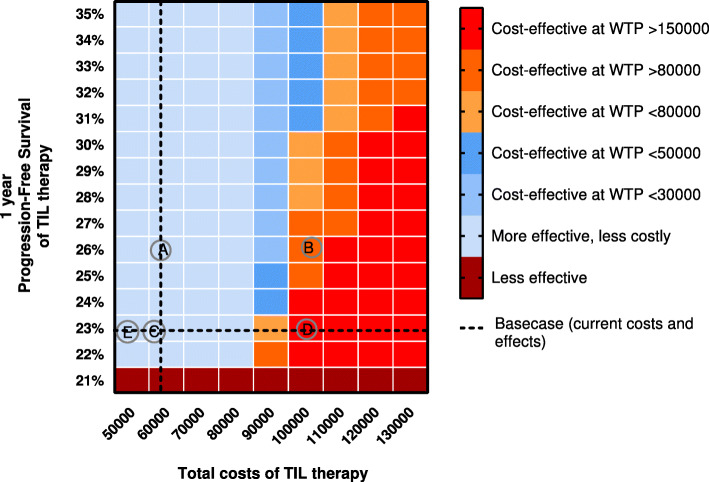
Two-way sensitivity analysis with visualization of the incorporated scenarios. Caption: This cross table shows the levels of cost-effectiveness at different willingness to pay levels of TIL therapy compared to ipilimumab when the Progression Free Survival (PFS) rate after 1 year changes and the costs of TIL vary. The dotted line represents the base case analysis. The incorporated scenarios are represented by letters. **a** = “TIL more effective”, **b** = “Combination therapy”, **c** = “Less IL2 treatment”, **d**= “TIL production outsourced”, **e** = “Automatic TIL production”. The scenario “low-cost competition” was not possible to present in this graph because it affects the costs of ipilimumab instead of the costs of TIL therapy. The colors do not always correspond with the results in Figs. 3 and 4 because we evaluated the rounded numbers of costs and PFS rate

Figures 3 and 4 show the NMB and probability of TIL therapy being cost-effective. Four out of six adoption scenarios showed a positive NMB: “TIL more effective”, “Low-cost competition”, “Less IL-2 treatment” and “Automatic TIL production”, and a high probability of being cost-effective. Even when the total costs of the comparator (ipilimumab) are reduced with 20%, TIL therapy had a 55% chance to be cost-effective (“Low-priced competition”). In contrast, “Combination therapy” showed a negative NMB with an ICER of €151,520 per QALY based on the first clinical results [19], and when the production of TILs is outsourced, TIL therapy had a 0% likelihood to become cost-effective (“TIL production outsourced”). All the results from the cost-effectiveness analysis are presented per scenario in Supplement 4. Figure 5 shows the results of the two-way sensitivity analysis and incorporated scenarios. This graph shows for instance that the effectiveness should improve substantially when TIL production is being outsourced or TIL therapy is combined with another therapy.

The combination of “TIL more effective” and “Automatic TIL production” showed a positive NMB as it combined the two most favorable scenarios for TIL therapy (more effective and less expensive). The other two combinations related to research developments showed that a slight improvement for TIL therapy in response rates does not outweigh the extra costs when TIL production is commercialized (0% probability of TIL being cost-effective), which holds when TIL therapy becomes more automatic (11% probability of TIL being cost-effective). The combinations that focused on the combination of TIL therapy with a different therapy, showed a negative NMB in all combinations of scenarios. A potential reduction of the costs of TIL therapy seems however to have the highest impact on the probability of being cost-effective (from 12% in the base case to 28% in the combination of “combination therapy” and “TIL production outsourced”).

## Discussion

Although a number of aspects concerning TIL therapy are uncertain, our results show that TIL therapy remains a promising addition to the treatment landscape of advanced melanoma as most of the “likely” scenarios resulted in TIL therapy being cost-effective. One should, however, keep in mind that these results were based on the safety and efficacy results that are currently available (phase I/II trials) [4–7]. The ongoing RCT conducted at NKI and Herlev Hospital (NCT02278887) is expected to bring the evidence needed to decide on its therapeutic position and adopt TIL therapy as a standard treatment option in advanced melanoma.

### Implications for clinical practice

The results of the cost-effectiveness analysis showed in most of the preferred scenarios a high probability for TIL therapy to become cost-effective (55–99%) and they identify aspects that could facilitate the wide adoption of TIL therapy. For example, as the scenario “Automatic TIL production” showed the highest probability for TIL therapy to become cost-effective (99%), Research and Development should focus on optimizing the production process to facilitate potential upscaling and adoption of TIL therapy. In contrast, the scenarios showing a reduced chance for TIL therapy to become cost-effective, identify crucial contextual factors that should be considered when deciding to adopt TIL therapy. For example, outsourcing of the production of TILs may at first be expected to overcome known ATMP barriers as (i) inadequate financial support for the required investments, and manufacturing costs; (ii) a lack of regulatory knowledge, and (iii) challenging to upscale the production and (iiii) to comply with Good Manufacturing Practices [9, 23–26]. However, as a result of commercial pricing levels, assuming that the costs will be at least 3 times as high, this scenario resulted in a 0% probability for TIL therapy to become cost-effective. Following our analysis, assuming a WTP threshold of €80,000, the production costs of TIL may only increase 1.5 times (~€53,000) to be cost-effective compared to ipilimumab. Within this scenario, it should be kept in mind that the estimation of the commercial costs is uncertain. Especially because our assumption was based on the manufacturing costs in an academic setting and literature showed that commercial prices are mostly linked to what society would pay instead of its expected added value or of the actual manufacturing costs [27]. However, although the estimation is uncertain, the conclusion related to commercialization remains the same: Outsourcing could facilitate the implementation of TIL therapy, however, pricing agreements should be made with the commercial party to ensure cost levels that remain within the willingness to pay range of cost-effectiveness. In the US setting, TIL can be expected to be licensed and filed for FDA approval within the coming years, these insights are especially of interest to guide reimbursement decisions by insurance companies. An interesting scenario is the “Combination therapy” which showed a 12% probability of being cost-effective (ICER of €151,520), revealing that in this case either the treatment costs should decrease or the efficacy has to improve considerably. By automatizing the production of TILs, the probability of being cost-effective increased only to 28%. Therefore, when a combination of TIL therapy and for instance a certain checkpoint inhibitor seems promising, agreements on pricing with pharmaceutical companies for the combination therapy are necessary to remain within cost-effectiveness ranges.

The results on the questions related to future developments suggest that the adoption of TIL therapy may be hampered by the attitude of patients and clinicians. First of all, clinicians nowadays seem unconvinced to apply TIL therapy because of its perceived complexity and treatment intensity unless the therapy shows a 1-year survival rate of at least 61.3% (CI:55.2–67.5). Secondly, only a small proportion of the eligible patients seemed to be aware of TIL therapy as a treatment option. As the attitude of stakeholders and especially clinicians, is a known barrier for implementation of ATMPs, a pro-active information strategy in anticipation of this attitude is crucial when deciding to diffuse TIL therapy [24].

### Comparison of our findings with current literature in the context of an ATMP

Another barrier that ATMPs face in the translational pathway is the rapidly evolving field of immunotherapy [8, 9]. We therefore compared our results with the most recent developments described in the literature and most of the “likely” scenarios still seem to be in line. For example, several trials investigate a combination of TIL therapy and other targeted therapies: pretreatment with ipilimumab followed by TIL and IL-2 (NCT01701674) [19] or pretreatment with vemurafenib followed by TIL and IL-2 and followed by vemurafenib (NCT01659151) [28]. Besides, several trials investigate or investigated the effectiveness of a lower dose of IL-2 treatment [17, 29] (NCT02354690), and finally, research groups evaluate the optimal process of producing TILs, aiming to improve the efficacy of TIL therapy e.g. by enriching T cell products with neo-antigens [17, 30].

Some developments found in literature, however, were not incorporated. For instance, several studies evaluate different lymphodepleting preparative regimens such as total body irradiation (TBI) in combination with chemotherapy [17, 31]. It is currently unclear whether such a regimen would be applied soon, but this scenario could influence the cost-effectiveness as TBI (requiring autologous PSC support) would significantly increase the costs. Besides, a very likely scenario that is not incorporated in this analysis, is the use of TIL therapy in other tumors such as renal cell cancer, ovarian cancer, and colorectal cancer [32–35] (NCT01174121). This scenario should be kept in mind as it may facilitate the adoption of TIL by positively influence the clinicians’ attitude as clinical experience and exposure increases, and production costs may decrease. Furthermore, a recent literature review highlighted several potential agents (e.g. TIM3, GITR, OX40) that could be promising in treating advanced melanoma in the future [36]. Those agents are currently subject to the first phase I and II studies to evaluate their safety and efficacy [36]. Therefore, our study might have underestimated the likelihood of the competition scenario. However, available data on the efficacy and possible costs of those alternatives is too preliminary to incorporate these results in the cost-effectiveness analysis. When these agents are proven to be safe, effective, and more effective compared to TIL therapy, those new treatments could hamper the adoption of TIL therapy.

#### Observations from the scenario method and future directions

A wide range in the expected likelihood of the scenarios was identified (Fig. 2), which challenged the labeling process for likely and unlikely scenarios. This may be explained by several factors. First, when a respondent is not (yet) involved in the TIL therapy process, it is harder to have an opinion on the likelihood of these scenarios as theoretical models describe that some extent of experience is needed to evaluate the future adoption process [37]. Second, faced with uncertainty, respondents could be hesitant in choosing extreme options such as 0 and 100% likelihood. Finally, it is likely that the expected timing of these scenarios, if they are likely, differ across countries and hospitals as the adoption process and attitude towards TIL differs per site. The respondents originated from 12 different countries which could thus explain some of the wide ranges, as in one country a scenario may be likely in the coming 5 years (e.g. commercialization in the US) and in another country not at all.

Furthermore, we are aware that the scenarios labeled as “unlikely” and therefore not incorporated in the cost-effectiveness analysis, could still play a role in the adoption process of TIL therapy (e.g. biomarker development, possible dominance of T-cell receptor (TCR) gene therapy over TIL therapy, influence by companies and competition). These factors should not be neglected and it would be valuable to incorporate these in future decision-making processes.

Additionally, as the chance on the development of other types of cancer by using TIL therapy was thought to be 6.4% (CI:4.5–8.3%; Supplement 6) on average, clinical studies having a longer follow-up time than the current observational studies should evaluate the actual risk. When the risk is shown to be evident, it should be ethically discussed whether TIL treatment may still be preferred over ipilimumab. Finally, based on the currently available clinical evidence, data are lacking for one of the most likely scenarios, “third-line treatment”, to evaluate the relative cost-effectiveness of TIL therapy. When estimating the expected costs of palliative chemotherapy we can estimate the incremental QALYs needed to become cost-effective at a certain willingness to pay threshold. When estimating the costs of on average 3.5 doses of chemotherapy (dacarbazine) [38] at €17.102 based on a three-weekly dosage of 200 mg/m2 for 5 days [20, 39] compared to the costs of TIL therapy, the difference in QALY’s should be at least 0.561. This means that TIL therapy has to show a substantial gain in survival and/or quality of life or a reduction in follow-up costs to become cost-effective in the third line. Such a calculation is informative but to inform decision-makers on the effects of this likely scenario, clinical outcomes after progression on both PD-1 inhibitors and CTL-4 antibodies based on e.g. clinical registries should be obtained. Next, we should compare these to clinical outcomes of TIL therapy in patients that progressed on multiple treatment strategies, such as reported in the study of Sarnaik and colleagues [40].

### Strengths and limitations

The main strength of the present study is that we systematically drafted future scenarios (qualitatively) with internal and external experts, using a Constructive Technology Assessment framework [11, 41] and using multiple Delphi rounds. This provides a comprehensive insight into the potential future developments that could influence TIL adoption and provides research and development teams with valuable information to anticipate possible future developments. Since the landscape of immunotherapy for melanoma is continuously developing, the expectations of the experts were compared to the most recent literature reviews and ongoing clinical trials to select the “likely” scenarios and discuss our results. The main limitation is obviously, that the scenarios may not even keep up with actual developments. Other limitations are related to the early nature of this analysis. For example, to simulate the combination therapy, the input for the model was based on a first observational study in which only 13 patients were enrolled that received the combination of TIL therapy and ipilimumab [19]. Additionally, the chosen cut-off value of ≥55% to evaluate scenarios as “likely” could be questioned due to the high uncertainty surrounding the likelihood scores. However, since the expert opinions and recent literature verified that the “likely” scenarios based on the cut-off value were “likely”, a different cut-off value is not expected to have altered our conclusions. Furthermore, the cost-effectiveness analyses were conducted from a Dutch perspective similar to the original model by Retèl et al. 2018. The costs for both TIL therapy and ipilimumab are expected to differ between countries [42] which limits the generalizability of the cost-effectiveness results in different settings. The generalizability may also be limited by the fact that mainly experts from European countries completed the questionnaire. However, by verifying the likelihood results with the most recent literature, the identified crucial contextual factors are expected to hold also in other countries because similar (financial) challenges are expected regarding e.g. outsourcing and providing a combination of therapies.

## Conclusion

The results of our scenario study can support the implementation and adoption process of TIL therapy as they identified crucial contextual factors that require anticipation and identified potential facilitators (e.g. commercialization of TIL therapy and a combination therapy). As implementation of TIL therapy is complex and could be time-consuming, clinicians and/or other decision-makers may decide to adapt the implementation process to possible developments in an early stage to anticipate and grant timely patient access when TIL therapy shows to be effective.

## Supplementary information

**Additional file 1: Supplement 1.** Questionnaire. **Supplement 2.** Identifying the “likely” scenarios. **Supplement 3.** Information on the input parameters for the base case analysis. **Supplement 4.** Full results of incorporation of the “likely” scenarios. **Supplement 5.** Reasoning for labelling scenarios likely or unlikely. **Supplement 6.** Results from the questions included in the questionnaire

## Data Availability

The datasets used and/or analyzed during the current study are available from the corresponding author on reasonable request.

## Abbreviations

ATMP: Advanced Therapy Medicinal Product
CED: Coverage with Evidence Development
CTL4: Cytotoxic T-Lymphocyte-associated protein 4
HTA: Health Technology Assessment
ICER: Incremental Cost-Effectiveness Ratio
IL-2: Interleukin-2
NMB: Net Monetary Benefit
NKI: Netherlands Cancer Institute – Antoni van Leeuwenhoek Hospital
PD1: Programmed cell death protein 1
QALY: Quality Adjusted Life Year
RCT: Randomized Controlled Trial
TA: Technology Assessment
TBI: Total Body Irradiation
TCR: T-cell Receptor therapy
TIL: Tumor Infiltrating lymphocytes
WTP: Willingness To Pay
