# Supplementary material for: Evaluating different adoption scenarios for TIL-therapy and the influence on its (early) cost-effectiveness
Source: BMC Cancer. 2020 Jul 31;20:712. doi: 10.1186/s12885-020-07166-9 (PMC7393723; doi:10.1186/s12885-020-07166-9)
Supplement: Supplementary file 1 — Additional file 1: Supplement 1. Questionnaire. Supplement 2. Identifying the “likely” scenarios. Supplement 3. Information on the input parameters for the base case analysis. Supplement 4. Full results of incorporation of the “likely” scenarios. Supplement 5. Reasoning for labelling scenarios likely or unlikely. Supplement 6. Results from the questions included in the questionnaire [file 12885_2020_7166_MOESM1_ESM.docx]

## Supplements

Supplement 1

Questionnaire

Supplement 2

Identifying the “likely” scenarios

Supplement 3

Information on the input parameters for the base case analysis

Supplement 4

Full results of incorporation of the “likely” scenarios

Supplement 5

Reasoning for labelling scenarios likely or unlikely

Supplement 6

Results from the questions included in the questionnaire

### Supplement 1 – Questionnaire

Welcome to this short questionnaire in which we try to explore the future of the tumor infiltrating lymphocytes (TIL) treatment in advanced melanoma.

"An exploration of the future, is not to predict the future but to explore plausible futures and become aware of the uncertainties."

Scenario drafting - the way we explore the future - is performed in order to identify plausible **barriers and facilitators** and to estimate the **full potential of this treatment** by means of a cost-effective analysis. Filling in this questionnaire will take approximately 15 minutes.

TIL, as you may know, is a personalized treatment for IIIC and IV stage melanoma in which at least a resectable lesion of 2-3 cm is available. In this treatment a tumor is resected and will be transported to a production facility. In this lab, Tcells will be harvested and will grow approximately 4-5 weeks untill a number of a couple billion cells is reached. After this period of growth, the patient will receive the infusion product of TILs. In addition interleukin-2 is given to stimulate the TILs inside the body. Below a schematic display of the procedure.


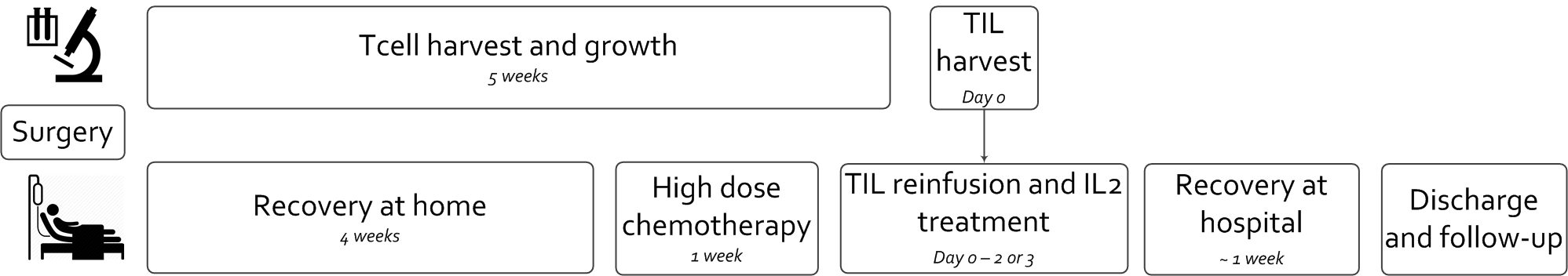


This questionnaire includes several questions and scenarios. For these scenarios we are interested in your estimation of the likelihood for this scenario to happen within 5 years.

Thank you for your time and effort!

With kind regards,

Melanie Lindenberg (m.lindenberg@nki.nl)

(on behalf of Prof. J. Haanen MD and Prof. W.H. van Harten MD)

**Part 1**

What is your function (work)? (open question)

To what company / hospital / institute are you affiliated to? (open question)

How many years of experience do you have with melanoma? (open question)

How many years of experience do you have with TIL? (open question)

To what extend do you find yourself familiar with the tumor infiltrating lymphocytes (TIL) treatment? (before the explanation that was given at the beginning of the questionnaire)

 Unfamiliar - When mentioning the subject, it does not recall any memories or it does not give lead to saying something sensible about it.

 Accidentally familiar - When you know what it is about, you have read something about it or you heard or saw something about it on the radio, television or other media.

 Familiar - When you know most arguments pro and contra the most controversial elements of the subject, when you have read a lot about it and when you have formed an opinion.

 Former expert - Used to be an expert on the subject some time ago, but our knowledge is somewhat outdated because other activities came up. But you are still reasonably well-informed about recent developments, which provides you with a broad overview of the subject as opposed to deep detailed knowledge.

Expert - When you belong to the small community of people who, at this moment, study, work on and are dedicated to this subject. You typically know who else works on this subject, you know the domestic literature and probably also the international literature about this subject, you go to conferences and seminars and when possible you publicize about the subject.

**Part 2**

**Background information**

Below, scenarios and questions are given. In these questions it is important to know the following definitions:

- Intervention of interest: TIL therapy
- Standard of care: ipilimumab
- Competing therapies: combination of therapies (ipilimumab and nivolumab or pembrolizumab), solely pembrolizumab, solely nivolumab or potential treatments currently in the pipeline

Furthermore, when italic text is given, this is extra information to help filling in the likelihood of the scenario or the question.

**Scoring scenarios**

0% I think this is not likely at all

50% This might as well, will or will not happen within 5 years

100% I am sure that this will be the case in 5 years

**Testcase**

Scenario: In the western countries, more than half of the car users will be using an electrical or hybrid car.

How likely is this scenario for the coming 5 years in percentages (0-100%)? (slider)

*TIL is part of a coverage with evidence development program in the Netherlands.*

**Scenario basecase 1**

If TIL shows better survival rates (at least 10% improvement) compared to ipilimumab, TIL will be reimbursed.

How likely is this scenario for the coming 5 years in percentages (0-100%)? (slider)

 I don't know

*TIL is part of a coverage with evidence development program in the Netherlands.*

**Scenario basecase 2**

If TIL shows better survival rates (at least 10% improvement) compared to ipilimumab, TIL will be implemented in specialized melanoma centers.

How likely is this scenario for the coming 5 years in percentages (0-100%)? (slider)

 I don't know

**Scenario effectiveness 1**

Competing (immuno)therapies are equal in costs but 10% more effective compared to TIL.

How likely is this scenario for the coming 5 years in percentages (0-100%)? (slider)

 I don't know

**Question effectiveness 1**

*Effectiveness of TIL Is likely to improve due to research developments such as gene modification or selective TIL, currently the one year survival rate is: 45% - 60% (based on phase 1 and 2 trials).*

What would be the minimal effectiveness of TIL leading to accept TIL as a standard therapy for you, expressed in one-year survival rate? (%)

**Scenario effectiveness 2**

The effectiveness of TIL has increased with 10% (clinically relevant) due to research developments.

How likely is this scenario for the coming 5 years in percentages (0-100%)? (slider)

 I don't know

**Question effectiveness 2**

What would be the risk of developing other types of cancer such as lymphomas by activating the immune system by injecting TILs? (in %)

**Question effectiveness 3**

*TIL is known as an intensive treatment, but compared to PD1 and CTL-4 inhibitors it may be argued that it is less intensive on longer term. First, the duration of TIL treatment is short compared to the other treatments: 3 weeks of hospital admission vs. 2-3 weekly sessions for a longer time period (ranging between 12 weeks and 1-2 years). Secondly, adverse events of TIL treatment are reversible and thus of a short duration, whereas competing therapies show substantial chances on high (long-term) toxicities (20%-40%).*

In what extend do you agree with the following statement:

TIL treatment provides significantly better quality of life compared to ipilimumab.

 Strongly agree

 Agree

 Nor agree or disagree

 Disagree

 Strongly disagree

*Currently a lot of research is focused on identifying factors that can predict a response on immunotherapy.*

**Scenario research 1**

A biomarker, being able to select patients for TIL, is available.

How likely is this scenario for the coming 5 years in percentages (0-100%)? (slider)

 I don't know

*T-cell receptor (TCR) gene therapies are currently in development in treating several types of cancer.*

**Scenario research 2**

TCR therapy dominates TIL treatment in advanced melanoma, regardless other treatment modalities.

 How likely is this scenario for the coming 5 years in percentages (0-100%)? (slider)

 I don't know

**Scenario patient perspective 1**

Patients prefer the competing therapies over TIL based on complete information on toxicities and effectiveness.

How likely is this scenario in the coming 5 years? (0-100%) (slider)

 I don't know

**Question patient perspective 1**

In the RCT TIL vs ipilimumab, patient recruitment is going slow. We think this is partly because of limited awareness of patients for this treatment.

Could you estimate the percentage of the eligible patients (metastatic melanoma patient) you think is aware of TIL as a potential treatment? (in %)

*Literature suggests that TIL is still effective after treatment with PD1 inhibitors.*

**Scenario implementation 1**

TIL is implemented as a second line treatment after anti PD1 inhibitors in metastatic melanoma.

How likely is this scenario for the coming 5 years? (0-100%) (slider)

 I don't know

**Scenario implementation 2**

TIL is implemented as a third line (last resort) treatment in metastatic melanoma.

How likely is this scenario for the coming 5 years? (0-100%) (slider)

 I don't know

**Scenario implementation 3**

TIL is used in combination with other immune or personalized therapies (i.e. nivolumab or vemurafenib).

How likely is this scenario for the coming 5 years? (0-100%) (slider)

 I don't know

**Question implementation**

If TIL proves to be effective, what would be the main reason for clinicians to be unconvinced of introducing TIL therapy in the coming 5 years?

 Complexity of TIL (understanding of TIL growth and the clinical process)

 Intensiveness of IL2 and expected adverse events

 User-friendliness of TIL (practical issues in implementation)

 This is not the case: clinicians will treat patients with TIL

 Other namely:

 

**Scenario implementation 4**

Clinicians are not willing to implement TIL because of one of the previous stated reasons.

How likely is this scenario for the coming 5 years in percentages (0-100%)? (slider)

 I don't know

**Scenario interactions with the pharmaceutical market 1**

If TIL turns out to be cost-effective, pharmaceutical companies will lower the prices of competing immunotherapies.

How likely is this scenario for the coming 5 years in percentages (0-100%)? (slider)

 I don't know

**Scenario interactions with pharmaceutical market 2**

Arrangements between pharmaceutical companies and hospitals and/or doctors, negatively affect patient selection for TIL therapy.

How likely is this scenario for the coming 5 years in percentages (0-100%)? (slider)

 I don't know

**Scenario process changes 1**

Additional interleukin-2 treatment after infusion of TIL is not be necessary anymore.

How likely is this scenario for the coming 5 years in percentages (0-100%)? (slider)

 I don't know

**Scenario process changes 2**

Production of TIL is of interest for the pharmaceutical market and is outsourced by a commercial company.

How likely is this scenario for the coming 5 years in percentages (0-100%)? (slider)

 I don't know

**Scenario process changes 3**

Production of TIL is less expensive (30% reduction) due to more automatic process steps.

How likely is this scenario for the coming 5 years in percentages (0-100%)? (slider)

 I don't know

This is the end of the questionnaire, thank you for your time and effort!

With kind regards,

Melanie Lindenberg

m.lindenberg@nki.nl

### Supplement 2 – Identifying the “likely” scenarios


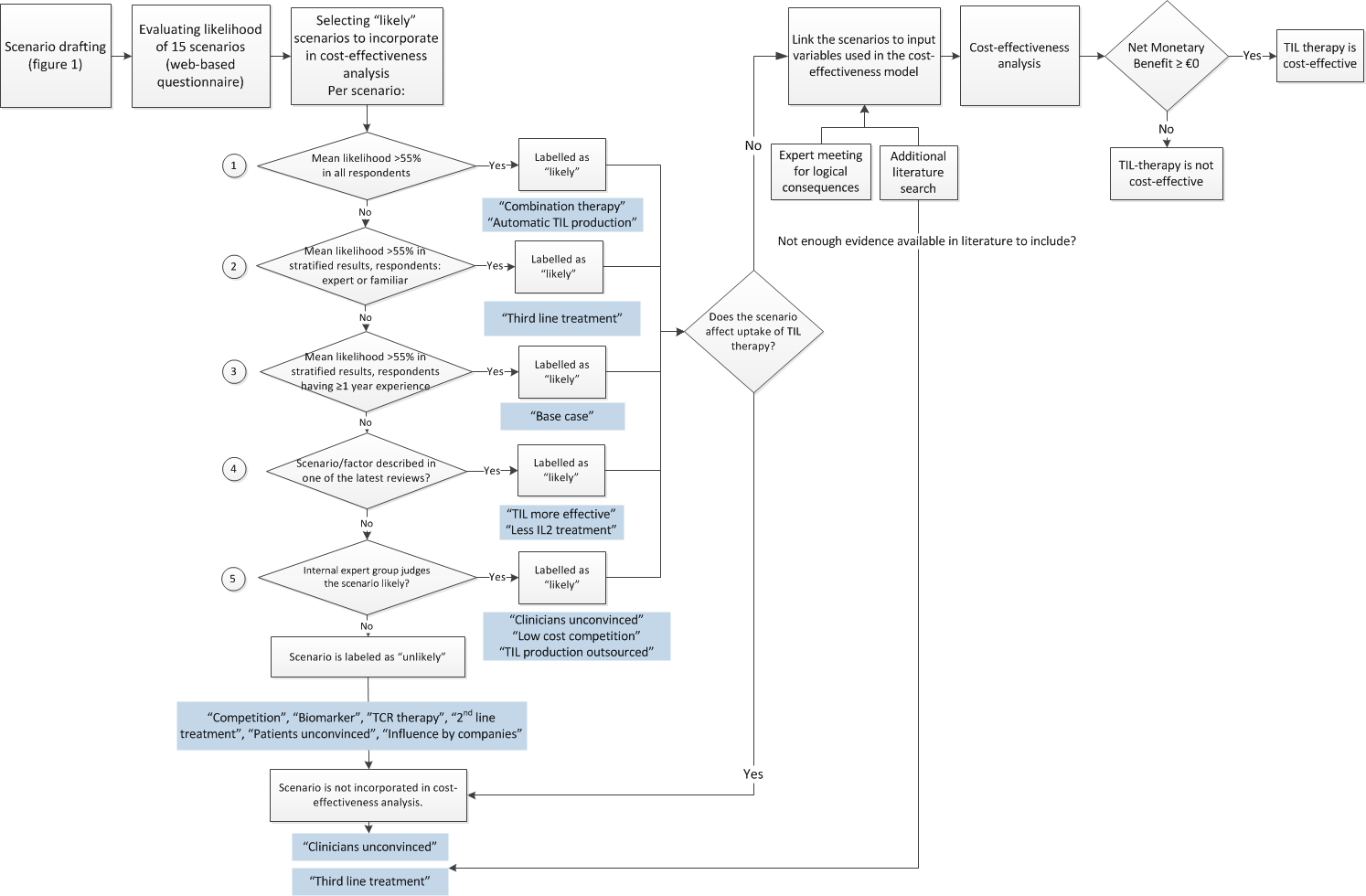


Supplement 2. Shows the process to selecting the “likely” scenarios that are incorporated in the cost-effectiveness analysis. The blue boxes describe which scenarios were labelled as “likely” after which step. For example: “TIL more effective” was not selected based on the results from the survey or the stratified results from the survey but selected because this theme was discussed in recent literature reviews.

### Supplement 3 – Information on the input parameters for the base case analysis

The original analysis by Retèl et al. 2018 presents a full overview of the methods for the base case analysis (1). In this supplement we provide data corresponding to this analysis.

**Data on progression free and overall survival**

The survival parameters were based on two studies describing both progression free and overall survival one year after TIL infusion. Besser et al. 2010 and Radvanyi et al. 2012 (2,3). Below the Kaplan Meier curves from the study by Radvanyi et al. 2012 are presented which are included as supplementary material. We did not adapted those graphs. The input for the pooled survival analysis using CMA Software, version 3, Biostat, US, was based on the two tables included in the manuscripts describing the months of progression free survival and overall survival.


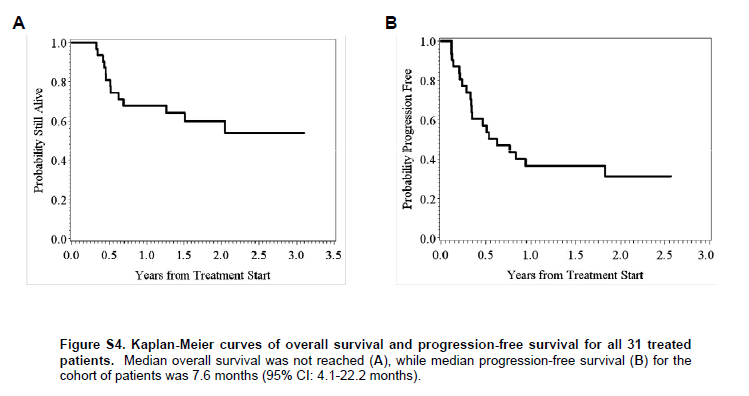


The input for the pooled analysis was as follows:

| Study | Events | Sample size | Event rate at one year | Standard error |
| --- | --- | --- | --- | --- |
| Progression free survival | | | | |
| Besser 2010 | 3 | 20 | 0.150 | 0.626 |
| Radvanyi 2012 | 10 | 31 | 0.323 | 0.384 |
| Overall survival | | | | |
| Besser 2010 | 7 | 20 | 0.350 | 0.469 |
| Radvanyi 2012 | 20 | 31 | 0.645 | 0.375 |

This resulted in the following pooled results for OS and PFS after 1 year. These rates were transformed in transition probabilities assuming a linear distribution. Those are presented in the next table.

|  | Pooled event rate | Lower limit | Upper limit |
| --- | --- | --- | --- |
| PFS | 0.266 | 0.160 | 0.408 |
| OS | 0.531 | 0.389 | 0.667 |

1. Retel VP, Steuten LMG, Geukes Foppen MH, Mewes JC, Lindenberg MA, Haanen JBAG, et al. Early cost-effectiveness of tumor infiltrating lymphocytes (TIL) for second line treatment in advanced melanoma: a model-based economic evaluation. BMC Cancer. 2018 Sep;18(1):895.

2. Besser MJ, Shapira-Frommer R, Treves AJ, Zippel D, Itzhaki O, Hershkovitz L, et al. Clinical responses in a phase II study using adoptive transfer of short-term cultured tumor infiltration lymphocytes in metastatic melanoma patients. Clin Cancer Res. 2010;

3. Radvanyi LG, Bernatchez C, Zhang M, Fox PS, Miller P, Chacon J, et al. Specific lymphocyte subsets predict response to adoptive cell therapy using expanded autologous tumor-infiltrating lymphocytes in metastatic melanoma patients. Clin Cancer Res. 2012;

**All input parameters**

This table shows the input parameters of the base case model. The table is copied from the original manuscript. The sources are listed below the table.

| **Parameters** | **Mean** | | **SE** | | **Distribution** | | **Source** | |
| --- | --- | --- | --- | --- | --- | --- | --- | --- |
| Survival probabilities per year | | | | | | | | |
| Ipilimumab |  | |  | |  | |  | |
| PFS | 0.175 | | 0.012 | | Beta | | [6] | |
| OS | 0.366 | | 0.018 | | Beta | | [6] | |
| TIL |  | |  | |  | |  | |
| PFS | 0.234 | | 0.089* | | Beta | | [10, 11] | |
| OS | 0.412 | | 0.098* | | Beta | | [10, 11] | |
| Utilities and side effects | | | | | | | | |
| Ipilimumab |  | |  | |  | |  | |
| Stable disease | 0.850 | | 0.020 | | Beta | | [18] | |
| Progression | 0.590 | | 0.020 | | Beta | | [18] | |
| TIL |  | |  | |  | |  | |
| Stable disease | 0.850 | | 0.020 | | Beta | | [18] | |
| Progression | 0.590 | | 0.020 | | Beta | | [18] | |
| Utility decrements |  | |  | |  | |  | |
| Fatigue | 0.090 | | 0.020 | | Beta | | [18] | |
| Diarrhea | 0.060 | | 0.020 | | Beta | | [18] | |
| Colitis | 0.130 | | 0.020 | | Beta | | [18] | |
| Neutropenia | 0.130 | | 0.020 | | Beta | | [18] | |
| Dyspnea | 0.100 | | 0.020 | | Beta | | [18] | |
| Flu-like syndrome (grade I/II)) | 0.090 | | 0.020 | | Beta | | [18] | |
| Anaemia | 0.110 | | 0.020 | | Beta | | [18] | |
| Likelihood of side effects |  | |  | |  | |  | |
| Ipilimumab |  | |  | |  | |  | |
| Fatigue | 0.070 | | 0.015 | | Beta | | [6] | |
| Diarrhea | 0.060 | | 0.015 | | Beta | | [6] | |
| Colitis | 0.060 | | 0.015 | | Beta | | [6] | |
| Dyspnea | 0.040 | | 0.015 | | Beta | | [6] | |
| Immune | 0.100 | | 0.015 | | Beta | | [6] | |
| Anaemia | 0.030 | | 0.015 | | Beta | | [6] | |
| TIL* |  | |  | |  | |  | |
| Fatigue | 0.001 | | 0.001 | | Beta | | [24] | |
| Diarrhea | 0.001 | | 0.001 | | Beta | | [24] | |
| Neutropenia | 0.560 | | 0.100 | | Beta | | [24] | |
| Dyspnea | 0.020 | | 0.015 | | Beta | | [24] | |
| Immune | 0.220 | | 0.100 | | Beta | | [24] | |
| Anaemia | 0.440 | | 0.100 | | Beta | | [24] | |
| Failures, non-compliance TIL | | | | | | | | |
| Failures | 0.100 | | 0.015 | | Beta | | | [20], Expert opinion |
| Non-compliance | 0.100 | | 0.015 | | Beta | | | [21] |
| Costs in euros | | | | | | | | |
| Ipilimumab-total | | 91,487.50 | | +/-25% | | Gamma | |  |
| Drug | | 90,100.00 | | +/-25% | | Gamma | | [22] |
| Administration | | 473.00 | | +/-25% | | Gamma | | [23] |
| Management of side effects | | 914.50 | | +/-25% | | Gamma | | [6, 16] |
| TIL** | | 62,000.00 | | +/-25% | | Gamma | | NKI-AVL |
| Interleukin treatment within the TIL therapy regimen | | € 2,130.24 | | +/-25% | | Gamma | | NKI-AVL and [22] |
| Follow-up costs stable disease*** | | 516.00 | | +/-25% | | Gamma | | [25] |
| Costs progressive disease**** | | 9,125.00 | | +/-25% | | Gamma | | [31] |
| Side effects | |  | |  | |  | |  |
| Fatigue | | 198.00 | | +/-25% | | Gamma | | [16] |
| Diarrhea | | 580.00 | | +/-25% | | Gamma | | [16] |
| Colitis/neutropenia***** | | 1115.00 | | +/-25% | | Gamma | | [16] |
| Dyspnea | | 100.00 | | +/-25% | | Gamma | | Assumption |
| Immune | | 7,680.00 | | +/-25% | | Gamma | | [16] |
| Anaemia | | 898.00 | | +/-25% | | Gamma | | [16] |

* Modeled in the first cycle of “stable disease”
**TIL costs including management of side effects, production costs and hospitalization
***based on 4* follow-up visit physician+CT scan (stable)
****cost for palliative care or end-stage disease care was based on the per diem cost of a palliative care unit
*****resembles 2-5 days hospitalization for severe toxicity (grade III-IV)
PFS: Progression Free Survival, OS: Overall Survival; SE: Standard Error
Input cost price calculation NKI-AVL: based on *N*= 10 patients from the pilot study
Inclusion criteria of the pilot study were: a resectable metastasis of at least 2-3cm; a sufficient heart, lung and kidney function; a maximum of 2 asymptomatic brain metastasis smaller than 1cm; not concurrently being treated with immune function-suppressing medication; not having auto-immune disorders; and a minimum expected life span of 3 months.

**References corresponding to the table.**

6. Hodi FS, O'Day SJ, McDermott DF, Weber RW, Sosman JA, Haanen JB, et al. Improved survival with ipilimumab in patients with metastatic melanoma. N Engl J Med. 2010;363:711–723. doi: 10.1056/NEJMoa1003466.

10. Besser MJ, Shapira-Frommer R, Treves AJ, Zippel D, Itzhaki O, Hershkovitz L, et al. Clinical responses in a phase II study using adoptive transfer of short-term cultured tumor infiltration lymphocytes in metastatic melanoma patients. Clin Cancer Res. 2010;16:2646–2655. doi: 10.1158/1078-0432.CCR-10-0041.

11. Radvanyi LG, Bernatchez C, Zhang M, Fox PS, Miller P, Chacon J, et al. Specific lymphocyte subsets predict response to adoptive cell therapy using expanded autologous tumor-infiltrating lymphocytes in metastatic melanoma patients. Clin Cancer Res. 2012;18:6758–6770. doi: 10.1158/1078-0432.CCR-12-1177.

16. National Institute for Health and Care Excellence (NICE). Ipilimumab for previously treated advanced (unresectable or metastatic) melanoma (TA268). In; 2012.

18. Beusterien KM, Szabo SM, Kotapati S, Mukherjee J, Hoos A, Hersey P, et al. Societal preference values for advanced melanoma health states in the United Kingdom and Australia. Br J Cancer. 2009;101:387–389. doi: 10.1038/sj.bjc.6605187.

20. Besser MJ, Shapira-Frommer R, Itzhaki O, Treves AJ, Zippel DB, Levy D, et al. Adoptive transfer of tumor-infiltrating lymphocytes in patients with metastatic melanoma: intent-to-treat analysis and efficacy after failure to prior immunotherapies. Clin Cancer Res. 2013;19:4792–4800. doi: 10.1158/1078-0432.CCR-13-0380.

21. Geukes Foppen MH, Donia M, Svane IM, Haanen JB. Tumor-infiltrating lymphocytes for the treatment of metastatic cancer. Mol Oncol. 2015;9:1918–1935. doi: 10.1016/j.molonc.2015.10.018.

22. Dutch National Health Care Institute. Dutch Pharmaceutical Guidelines, consulted in 2016; avaliable at: <https://www.medicijnkosten.nl/>.

23. Barzey V, Atkins MB, Garrison LP, Asukai Y, Kotapati S, Penrod JR. Ipilimumab in 2nd line treatment of patients with advanced melanoma: a cost-effectiveness analysis. J Med Econ. 2013;16:202–212. doi: 10.3111/13696998.2012.739226.

24. Ellebaek E, Iversen TZ, Junker N, Donia M, Engell-Noerregaard L, Met O, et al. Adoptive cell therapy with autologous tumor infiltrating lymphocytes and low-dose Interleukin-2 in metastatic melanoma patients. J Transl Med. 2012;10:169. doi: 10.1186/1479-5876-10-169.

25. Hakkaart-van Roijen L vdLN, Bouwmans C, Kanters T, Tan SS,. Kostenhandleiding. Methodologie van kostenonderzoek en referentieprijzen voor economische evaluaties in de gezondheidszorg. (in Dutch). In opdracht van Zorginstituut Nederland. 2015.

31. Crott, R. Cost effectiveness and cost utility of adjuvant interferon α in cutaneous melanoma: A review. *PharmacoEconomics* (2004). doi:10.2165/00019053-200422090-00002

### Supplement 4 – Results from the cost-effectiveness analysis per incorporated scenario

|  | | | | | **# patients in the model** | | | **Costs** | | **QALYs** | **LYs** | | **iCosts** | | | **iQALYs** | | | **ICER** | | **Conclusion** | **NMB** | | | **Probability of TIL being cost-effective** | | | | | |
| --- | --- | --- | --- | --- | --- | --- | --- | --- | --- | --- | --- | --- | --- | --- | --- | --- | --- | --- | --- | --- | --- | --- | --- | --- | --- | --- | --- | --- | --- | --- |
| **BASECASE RESULTS** (1) | | | | | | | | | | | | | | | | | | | | | | | | | | | | | | |
| TIL | | | | | 1000 | | | € 81,085 | | 0.43 | 0.68 | |  | | |  | | |  | |  |  | | |  | | | | | |
| Ipilimumab | | | | | 1000 | | | € 94,705 | | 0.38 | 0.58 | |  | | |  | | |  | |  |  | | |  | | | | | |
|  | | | | |  | | |  | |  |  | | -€ 13,620 | | | 0.050 | | | -€ 270,281 | | DOMINANT | € 19,473 | | | 89% | | | | | |
| **Scenarios** | | | | | | | | | | | | |  |  | | |  | | | | | | |  | | | | | |  |
| **“Base case” -** If TIL shows better survival rates (at least 10% improvement) compared to ipilimumab, TIL will be implemented. | | | | | | | | | | | | | | | | | | | | | | | | | | | | | | |
| TIL | | | | | | 46/58* | €81,085 | | | 0.43 | 0.68 | |  | | |  | | |  | |  |  | | | |  | | | | |
| Ipilimumab | | | | | | 89 | €94,705 | | | 0.38 | 0.58 | |  | | |  | | |  | |  |  | | | |  | | | | |
|  | | | | | |  |  | | |  |  | | -€13,620 | | | 0.050 | | | -€270,281 | | DOMINANT | €19,693 | | | | 90% | | | | |
| **“TIL more effective” -** The effectiveness of TIL has increased with 10% (clinically relevant) due to research developments. | | | | | | | | | | | | | | | | | | | | | | | | | | | | | | |
| TIL | | | | 1000 | | | € 81,668 | | | 0.49 | | 0.78 |  | |  | | |  | |  | | |  | | |  | | | | |
| Ipilimumab | | | | 1000 | | | € 94,705 | | | 0.38 | | 0.58 |  | |  | | |  | |  | | |  | | |  | | | | |
|  | | | |  | | |  | | |  | |  | -€ 13,037 | | 0.114 | | | -€114,128 | | DOMINANT | | | € 23,270 | | | 92% | | | | |
| **“Combination therapy”** - TIL is used in combination with other immune or personalized therapies (i.e. nivolumab or vemurafenib) | | | | | | | | | | | | | | | | | | | | | | | | | | | | | | |
| TIL | | | | 1000 | | | | | € 123,853 | 0.57 | 0.91 | |  | |  | | |  | |  | |  | | | |  | | | | |
| Ipilimumab | | | | 1000 | | | | | € 94,705 | 0.38 | 0.58 | |  | |  | | |  | |  | |  | | | |  | | | | |
|  |  | | |  | | | | |  |  |  | | € 29,148 | | 0.192 | | | € 151,520 | | DOMINATED | | -€ 10,837 | | | | 12% | | | | |
| **“Low cost competition”** - If TIL turns out to be cost-effective, pharmaceutical companies will lower the prices of competing immunotherapies. | | | | | | | | | | | | | | | | | | | | | | | | | | | | | | |
| TIL | | | | 1000 | | | | | € 77,478 | 0.43 | | 0.68 |  | |  | | |  | |  | |  | | | | |  | | | |
| Ipilimumab | | | | 1000 | | | | | € 75789 | 0.38 | | 0.58 |  | |  | | |  | |  | |  | | | | |  | | | |
|  |  | | |  | | | | |  |  | |  | € 1,512 | | 0.050 | | | € 30,014 | | Threshold | | € 11,418 | | | | | 56% | | | |
| **“Less IL2 treatment”** - Additional interleukin-2 treatment after infusion of TIL is not be necessary anymore. | | | | | | | | | | | | | | | | | | | | | | | | | | | | | | |
| TIL | | | | 1000 | | | | | € 80,590 | 0.43 | | 0.68 |  | |  | | |  | |  | |  | | | | |  | | | |
| Ipilimumab | | | | 1000 | | | | | € 94,705 | 0.38 | | 0.58 |  | |  | | |  | |  | |  | | | | |  | | | |
|  | |  | |  | | | | |  |  | |  | -€ 14,115 | | 0.049 | | | -€ 287058 | | DOMINANT | | € 20366 | | | | | 92% | | | |
| **“TIL production outsourced”** - Production of TIL is of interest for the pharmaceutical market and is outsourced by a commercial company. | | | | | | | | | | | | | | | | | | | | | | | | | | | | | | |
| TIL | | | | 1000 | | | | | € 152,085 | 0.43 | | 0.68 |  | |  | | |  | |  | |  | | | | | | |  | |
| Ipilimumab | | | | 1000 | | | | | € 94,705 | 0.38 | | 0.58 |  | |  | | |  | |  | |  | | | | | | |  | |
|  | | |  |  | | | | |  |  | |  | 57,380 | | 0.050 | | | € 1,138,642 | | DOMINATED | | -€ 51,551 | | | | | | | 0% | |
| **“Automatic TIL production”** - Production of TIL is less expensive (30% reduction) due to more automatic process steps. | | | | | | | | | | | | | | | | | | | | | | | | | | | | | | |
| TIL | | | | 1000 | | | | | € 70,435 | 0.43 | | 0.68 |  | |  | | |  | |  | |  | | | | | |  | | |
| Ipilimumab | | | | 1000 | | | | | € 94,705 | 0.38 | | 0.58 |  | |  | | |  | |  | |  | | | | | |  | | |
|  | | |  |  | | | | |  |  | |  | -€ 24,270 | | 0.050 | | | -€ 481,620 | | DOMINANT | | € 22,667 | | | | | | 99% | | |

### Supplement 5 – Reasons for in and excluding scenarios for cost-effectiveness modelling

| **Scenario** | **Labelled as** | **Reason** |
| --- | --- | --- |
| “Base case” | Likely | The mean likelihood in respondents having 1 or more years of experience was above 55%. (Figure 2) Besides, the respondents described a minimal 1-year survival of 61.3% (CI: 55.2%-67.5%) to be the minimal acceptable effectiveness of TIL therapy to adopt it, which is shown by the published results from the observational studies. |
| “Competition” | Unlikely | This scenario was discussed in the internal research group. In the literature review used for further selection, no medicines were described to be in development that are expected to be more effective and/or equal in costs than TIL therapy(6). This could however be explained by the scope of the review. |
| “TIL more effective” | Likely | In the review used to identify likely scenarios several developments were discussed that would result in TIL therapy being more effective(6). |
| “Biomarker” | Unlikely | A biomarker to specifically select patients that would benefit from TIL therapy seems not likely to be discovered in the coming years as similar variables seem to be prognostic for selecting patients for CTL-4 and PD1 antibodies(6). Also the expected mean likelihood of this scenario was scored below 40%, indicating that this scenario is unlikely to happen in the coming 5 years. |
| “TCR therapy” | Unlikely | Results of TCR gene therapy are still too preliminary and therefore it as unlikely that this treatment would become available and would dominate TIL therapy within 5 years (6,7). Also the expected mean likelihood of this scenario was scored below 40%, indicating that this scenario is unlikely to happen in the coming 5 years. |
| “Patients unconvinced” | Unlikely | This scenario was discussed in the internal research group. Based on a previous analysis in which we evaluated factors that may influence the choice of receiving TIL therapy among eligible patients, patients were positive about receiving TIL therapy (8). |
| “2^nd^ line treatment” | Unlikely | This scenario was discussed in the internal research group. This scenario is currently similar to the base case model which was already simulated. The addition of this scenario in the cost-effectiveness analysis would thus not add information.  Furthermore, in light of the developments and new treatment options available it is more likely that TIL therapy will be placed after progression on 1^st^ line treatment with anti-PD1 and 2^nd^ line treatment with CTL-4 antibodies. Therefore this scenario was labelled “unlikely”. |
| “3^rd^ line treatment” | Likely | The mean likelihood in respondents describing themselves as familiar or an experts and in respondents having 1 or more years of experience was above 55%. (Figure 2) |
| “Combination therapy” | Likely | The mean likelihood in all respondents was above 55%. |
| “Clinicians unconvinced” | Likely | This scenario was discussed in the internal research group. In the additional questions that were asked in the web-based questionnaire only 3 of 29 respondents described that none of the stated reasons (complexity, user-friendliness or intensity of IL2 treatment) applied to be unconvinced on the use of TIL therapy and that clinicians would apply TIL therapy. Besides, they expressed to be mainly indifferent (31%) or to disagree (37%) with the statement that TIL therapy would improve quality of life of patients compared to ipilimumab.  Therefore it was thought very likely that clinicians remain unconvinced in the coming years. |
| “Low cost competition” | Likely | This scenario was discussed in the internal research group. Following the interests of pharma and trends seen in decreasing medicines it is thought to be very likely that pharma would lower the prices of competing immunotherapies when TIL therapy turns out to be a true and less expensive competitor. |
| “Less IL2 treatment” | Likely | This scenario was discussed in the literature review showing that several research group are evaluating different IL2 dosing schemes(6), therefore this scenario was labelled as likely. |
| “Influence by companies” | Unlikely | This scenario was discussed in the internal research group. By our clinical experts it is not expected that this would have a significant influence. If TIL therapy would be (cost-)effective, clinicians would provide this treatment regardless their agreements with pharmaceutical companies. |
| “TIL production outsourced” | Likely | This scenario was discussed in the internal research group. The head of the production facility explained that this scenario has been seen in other countries and therefore it seems a very realistic scenario. |
| “Automatic TIL production” | Likely | The mean likelihood in all respondents was above 55%. |

### Supplement 6 - Results from the questions included in the questionnaire

|  | |  | # Respondents (%) | | | Mean score (95% CI) |
| --- | --- | --- | --- | --- | --- | --- |
| Theme: effectiveness | | | | | | |
| What would be the minimal effectiveness of TIL leading to accept TIL as a standard therapy for you? Expressed in one-year survival rate (%)? | | | 26(89%) | | 61.3%  (55.2-67.5) | |
| What would be the risk of developing other types of cancer such as lymphomas by activating the immune system by injecting TILs (%)? | | | 24 (83%) | | 6.4%  (4.5 – 8.3) | |
| Agreement with statement: TIL treatment provides significantly better quality of life compared to ipilimumab. | | | 29 (100%) | |  | |
|  | Strongly agree  Agree  Nor agree or disagree  Disagree  Strongly disagree | | 2 (7%)  7 (24%)  9 (31%)  10 (34%)  1 (3%) | | | |
| Theme: Patient perspective | | | | | | |
| Could you estimate the percentage of the eligible patients (metastatic melanoma patients) you thinks is aware of TIL as a potential treatment (in %) | | | 25 (86%) | | 20.8%  (14.7-26.9) | |
| Theme: Clinician’s attitude | | | | | | |
| What would be the main reason for clinicians to be unconvinced of introducing TIL therapy? (more options were possible) | | | 29 (100%) 47 answers | | | |
|  | Complexity of TIL (understanding of TIL growth and the clinical process)  Intensiveness of IL2 and expected adverse events  User-friendliness of TIL (practical issues in implementation)  This is not the case: clinicians will treat patients with TIL | | | 18/47 (38%)  15/47 (32%)  11/47 (23%)  3/47 (6%) | | |
